# Artificial intelligence in early diagnosis and prevention of oral cancer

**DOI:** 10.1016/j.apjon.2022.100133

**Published:** 2022-08-24

**Authors:** Shruthi Hegde, Vidya Ajila, Wei Zhu, Canhui Zeng

**Affiliations:** a Nitte (Deemed to Be University), AB Shetty Memorial Institute of Dental Sciences (ABSMIDS), Department of Oral Medicine and Radiology, Mangalore, India; b Department of Nursing, Shandong Medical College, Jinan, China; c Department of Emergency Medicine, Integrative Hospital of Traditional Chinese Medicine, Southern Medical University, Guangzhou, China

**Keywords:** Oral cancer, Artificial intelligence, Screening, Early diagnosis

## Abstract

The global occurrence of oral cancer (OC) has increased in recent years. OC that is diagnosed in its advanced stages results in morbidity and mortality. The use of technology may be beneficial for early detection and diagnosis and thus help the clinician with better patient management. The advent of artificial intelligence (AI) has the potential to improve OC screening. AI can precisely analyze an enormous dataset from various imaging modalities and provide assistance in the field of oncology. This review focused on the applications of AI in the early diagnosis and prevention of OC. A literature search was conducted in the PubMed and Scopus databases using the search terminology “oral cancer” and “artificial intelligence.” Further information regarding the topic was collected by scrutinizing the reference lists of selected articles. Based on the information obtained, this article reviews and discusses the applications and advantages of AI in OC screening, early diagnosis, disease prediction, treatment planning, and prognosis. Limitations and the future scope of AI in OC research are also highlighted.

## Introduction

The global occurrence of oral cancer (OC) has increased in recent years, with oral squamous cell carcinomas (OSCCs) counting for more than 90% of these cancers.^1^ OSCCs are also the sixth most common malignancy in the world. In 2012, The World Health Organization reported 529 ​000 new cases of OC and 300 ​000 deaths due to OC each year.^2^ OC that is diagnosed in its advanced stage results in morbidity and mortality. A crucial factor in providing successful treatment is the early detection of cancerous lesions. Inaccessible lesions and the late detection of cancers are associated with low survival, increased symptoms, and a higher treatment cost.^3^ Early diagnosis can increase the survival rate to 75%–90%.^1^^,^^4^

Early detection includes the diagnosis of oral potentially malignant disorders (OPMDs) and regular follow-ups. OPMDs have been defined as “any oral mucosal abnormality that is associated with a statistically increased risk of developing OC.”^5^ OPMDs include oral leukoplakia, proliferative verrucous leukoplakia, erythroplakia, oral lichen planus, oral submucous fibrosis, palatal lesions in reverse smokers, oral lupus erythematosus, actinic keratosis, and dyskeratosis congenita. Newly included lesions in the recent classification are oral lichenoid lesion and oral chronic graft-versus-host disease.^5^

Initial detection of OC requires self-examination of the oral cavity as well as professional consultation. Screening of high-risk populations is needed to avoid the late diagnosis, but these populations are often located in remote regions with limited access to healthcare facilities. Poor knowledge regarding OC symptoms is a major hindrance.^1^^,^^2^ The use of technology may be beneficial for the early detection of OC. The advent of artificial intelligence (AI) has the potential to improve OC screening.^2^ The increase in research based on AI technology for medical imaging and diagnosis has been promising. AI technologies have been found to be effective in detecting breast, lung, and oral cancers.^6^ The potential of AI to improve the efficiency of OC screening is the reason for its implementation in oncology.^6^

AI can be divided into traditional machine learning (ML) and deep learning. Traditional ML uses algorithms and computer processes to calculate information and recognize input data patterns to offer a quantified diagnostic result.^7^ ML methods are divided into supervised and unsupervised.^8^^,^^9^ Deep learning or neural networks are techniques comprising of nonlinear processing units with multiple layers to learn and understand input and associate output with the relevant input.^10^^,^^11^

Presently, these techniques are being assessed for more effective methods for diagnosis, especially for the screening of diseases, where fewer doctors and trained experts are available.^6^ AI can be used in many ways in the prevention of OC and its early diagnosis. AI can precisely analyze a vast dataset of various imaging modalities, such as fluorescent, hyperspectral, cytological, histological, radiological, endoscopic, clinical, and infrared thermal modalities.^12^ Recently, vision-based adjunctive technologies were developed to detect OPMDs that carry the risk of cancer development. This review discusses the applications of AI in OC screening, early diagnosis, disease prediction, treatment planning, and prognosis.

## Methods

This review was done in accordance with the Preferred Reporting Items for Systematic Reviews and Meta-Analyses (PRISMA) guidelines.^13^

### Literature search

A data search was carried out in two databases, namely PubMed and Scopus. The search string (“oral cancer” AND “Artificial intelligence”) was used to search both databases for articles published between 2012 and 2022. Further research was conducted to extract information regarding the topic based on the reference lists of the selected articles and pertinent reviews. Details about AI in OC screening, early diagnosis, prediction, and management were obtained from the articles that met the selection criteria for the present review.

### Inclusion and exclusion criteria

The inclusion criteria for the articles were as follows:

• Published between 2012 and 2022.
• Original research articles.
• Full text available in the institutional digital library.
• Written in English or Chinese.

The exclusion criteria were as follows:

• Animal studies.
• Types of study: Case reports, short communications, personal viewpoints, letters to editors, conference abstracts, and literature reviews.
• Not written in English or Chinese.

### Study selection and data extraction

The titles, abstracts, and full texts of the relevant articles were examined separately by two reviewers, and the consensus was obtained regarding each article's inclusion or exclusion. The reviewers retrieved the following data from the eligible articles: author, year, aims and objectives, sample type, methods, and study conclusions.

## Results

**Fig. 1:**
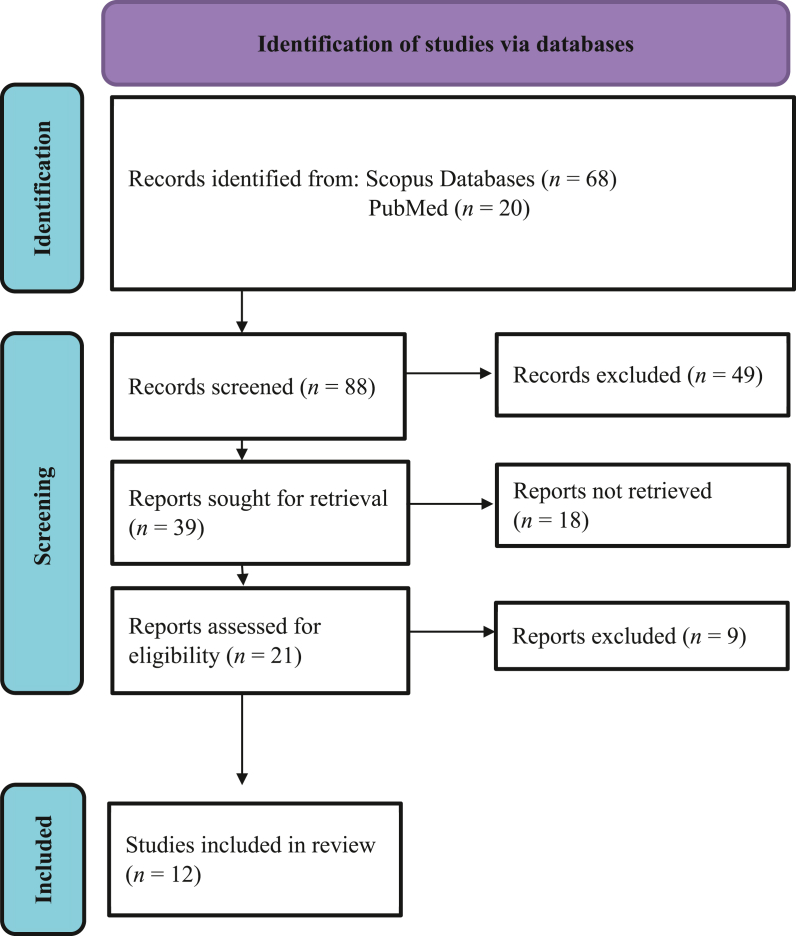
PRISMA flowchart.

The article selection process followed is documented in the PRISMA flowchart (Fig. 1). The literature search in the databases resulted in a total of 88 potential records. The title and abstracts of these records were evaluated in terms of the selection criteria, resulting in 49 exclusions. Reports were sought for retrieval from the remaining articles, of which the full text of 21 articles was read by the reviewers for potential inclusion. After this screening process, 12 articles were selected, and the data were retrieved, as shown in Table 1.^14^, ^15^, ^16^, ^17^, ^18^, ^19^, ^20^, ^21^, ^22^, ^23^, ^24^, ^25^

**Table 1:** Summary of studies using AI in oral cancer early detection, diagnosis, and treatment outcome.

Author (Year)	Aims	Sample	Methods	Main findings
Adeoye (2021)^24^	To compare and validate supervised deep and conventional learning algorithms for the risk-probability prediction of malignant transformation in OPMDs	A total of 716 patients with a clinical diagnosis of oral leukoplakia, oral lichen planus, or oral lichenoid lesions who underwent biopsy	Twenty-six features available from electronic health records were used to train four learning algorithms—Cox-Time, DeepHit, Deep Surv, and random survival forest	Time-to-event models are successful in predicting the malignant transformation of oral leukoplakia and oral lichenoid lesions.
Alhazmi (2021)^22^	To develop an artificial neural network model that helps to predict the individuals' risk of developing oral cancer based on data on risk factors, systematic medical condition, and clinic-pathological features	Seventy-three cases with confirmed diagnosis and pathologic reports were included in this study	A popular data mining algorithm artificial neural network was used for developing the artificial intelligence-based prediction model. A total of 29 variables that were associated with the patients were used for developing the model. The dataset was randomly split into the training dataset 54 (75%) cases and testing dataset 19 (25%) cases	Machine learning technique has the potential to help in oral cancer screening and diagnosis based on the datasets. The results demonstrate that the artificial neural network could perform well in estimating the probability of malignancy and improve the positive predictive value that could help to predict the individuals' risk of developing OC based on knowledge of their risk factors, systemic medical conditions, and clinic-pathological data.
Chu (2021)^23^	To revisit this well-characterized patient cohort to evaluate the ability of supervised machine learning models to predict disease outcome	Retrospective review of 467 OSCC patients treated over a 19-year period facilitated the construction of a detailed clinicopathological database	Overall survival was determined from the date of primary diagnosis until death or most recent clinic follow-up. Thirty-four prognostic features from the database were used to populate four machine learning algorithms, such as LR, DT, SVM, and KNN models, to attempt progressive disease outcome prediction	Machine learning models assist clinicians in accessing digitized health information and appear promising in predicting progressive disease outcomes.
James et al. (2021)^21^	To integrate OCT imaging with automated image processing and deep learning to reduce the subjectivity in image interpretation, and it is large-scale, in-vivo, validation in the delineation of OSCC, and dysplastic lesions from normal/benign lesions in both community and tertiary care settings.	Validation of a portable, robust OCT device in 232 patients (lesions: 347) in different clinical settings	OCT imaging was followed by incisional or excisional biopsy. The captured images were classified by a simple algorithm. The image features were extracted using multiple ANN and the SVM model. Both the methods were compared with histological or clinical diagnosis depending on whether biopsy was indicated or not	The study provides evidence toward the utility of the robust and low-cost OCT instrument as a point-of-care device in resource-constrained settings and the potential clinical application of device in screening and surveillance of oral cancer
Jubair et al. (2022)^15^	To develop a lightweight deep CNN for binary classification of oral lesions into benign and malignant or potentially malignant using standard real-time clinical images	716 clinical images	A small deep CNN that uses a pretrained EfficientNet-B0 as a lightweight transfer learning model	Deep CNNs can be an effective method to build low-budget embedded vision devices with limited computation power and memory capacity for the diagnosis of oral cancer.
Marzouk (2022)^19^	To introduce AI with deep transfer learning-driven oral cancer detection and classification model (AIDTLOCCM). The primary goal of the proposed AIDTLOCCM model is to diagnose oral cancer using AI and image processing techniques.	Sample images: cancer (87 images) and non-cancer (44 images)	AIDTLOCCM model involves a fuzzy-based contrast enhancement approach to perform data pre-processing. Followed by the densely connected networks model is employed to produce a useful set of deep features. Chimp optimization algorithm (COA) with autoencoder (AE) model is applied for oral cancer detection and classification. COA is employed to determine optimal parameters involved in AE model.	Enhanced performance of AIDTLOCCM model compared to other approaches with a maximum accuracy of 90.08%
Nguyen et al. (2022)^16^	To evaluate the potential of AI methods for histopathological grading of tongue dysplasia	A dataset comprising 203 digitized whole-slide images (WSIs) was constructed at 200 ​× ​magnification from surgical specimens of tongue dysplasia and early cancers	Convolutional neural network Inception-v3	A properly trained AI system has the potential to improve the accuracy of oral dysplasia grading on WSIs.
Rahman (2020)^25^	To identify OSCC based on morphological and textural features of hand-cropped cell nuclei by traditional machine learning methods	Forty biopsy slides were used for the study	Out of 45 biopsy slides, 452 hand-cropped cell nuclei have been considered for morphological and textural feature extraction and further analysis. After making a comparative analysis of commonly used methods in the segmentation technique, a combined technique is proposed.	Study concluded that both morphological and textural features play a very important role in OSCC diagnosis.
Sharma (2022)^18^	To use pre-trained convolutional neural networks for identifying oral pre-cancerous (potentially malignant) and cancerous lesions and to differentiate them from normal mucosa using a dataset of clinically annotated photographic images.	Clinical photographs of 329 oral squamous cell carcinoma and OPMDs patients. A comparative analysis of these photographs was done with photographs of normal oral mucosa. Oral cavity images acquired using smartphones	Pre-trained CNNs are used for identifying oral pre-cancerous and cancerous lesions and to differentiate them from normal mucosa using a dataset of clinically annotated photographic images	Study demonstrated the performance of CNN models in identification and classification comparable to a biopsy report
Sun et al. (2022)^17^	To validate a novel method to predict the proliferation status of TSCC using contrast-enhanced CT (CECT) based on AI	CECT images of the lesion area from 179 TSCC patients	Sample analyzed using a CNN	Study provided a possibility of predicting the proliferation status of TSCC using AI in CECT noninvasively before operation
Warin (2021)^20^	To use the CNN deep learning algorithms to develop an automated classification and detection model for oral cancer screening.	A total of 700 clinical oral photographs were collected retrospectively from the oral and maxillofacial center, which were divided into 350 images of oral squamous cell carcinoma and 350 images of normal oral mucosa	Classification and detection models were created by using DenseNet121 and faster R-CNN, respectively. Four hundred and ninety images were randomly selected as training data. In addition, 70 and 140 images were assigned as validating and testing data, respectively	The DenseNet121 and faster R-CNN algorithm were proved to offer the acceptable potential for classification and detection of cancerous lesions in oral photographic images.
Warin et al. (2022)^14^	To evaluate CNN algorithms and classify and detect OPMDs in oral photographs	A total of 600 oral photograph images (300 images of OPMDs and 300 images of normal oral mucosa)	CNN-based classification models were created using DenseNet-121 and ResNet-50. The detection models were created using faster R-CNN and YOLOv4	DenseNet-121, ResNet-50, and faster R-CNN models have potential for the classification and detection of OPMDs in oral photographs.

AI, artificial intelligence; ANN, artificial neural networks; CNN, convolutional neural network; DT, decision tree; LR, linear regression; KNN, k-nearest neighbors; OPMD, oral potentially malignant disorders; SVM, support vector machine; TSCC, tongue squamous cell carcinoma.

AI presents extensive opportunities in OC screening. Advances in the field of AI offer a powerful adjunctive method to perform an automated screening of the oral cavity. This would provide feedback to healthcare professionals during the examination of patients, as well as to individuals for self-examination.^1^ The various modalities used in the previous studies pertaining to AI and OC research are tabulated in Table 2. The vast amount of digitized data from AI can empower clinicians^12^; the applications and advantages of AI in oral oncology are indicated in Table 3.

**Table 2:** Datasets used in AI and oral cancer research.

• Clinical images and photographic images
• Patients' geographic data and habits history
• Autofluorescence image and white light image
• Optical coherence tomography
• Raman spectroscopy
• Spectroscopy probe
• Confocal laser endomicroscopy images
• Multidimensional hyperspectral images
• Gene expression data
• Radiographic images such as computed tomography (CT) images and magnetic resonance imaging (MRI)
• Saliva metabolites
• Histopathologic images and P 53 immunostained tissue section

**Table 3:** Applications and advantages of AI in oral oncology.

• Screening of high-risk populations
• Helps in early diagnosis of oral cancer in populations residing at remote regions with limited access to healthcare facility
• Ability to precisely analyze an enormous dataset
• Detection and classification of cancerous lesions
• Interpretation of images as normal oral mucosa/precancerous/cancerous lesions
• Ease of use in multicenter study
• AI allows automated learning without human arbitration
• Automate processes and combine variables at different levels and provide outcome
• Ability to constantly train on further data
• Guide clinician in decision-making
• AI system assists expert pathologist to deliver superior results with minimum diagnostic errors
• Potential for combination of history, geographical data, risk factors, clinicopathologic features, imaging features, and omics data to generate risk assessments
• Prediction of the malignant transformation of OPMDs
• Detecting accurate biomarkers
• Lymph node metastasis prediction
• Support clinician in treatment planning

## Discussion

### AI in oral cancer screening and detection

Recent systematic reviews reported that Asia had the highest incidence of lip and OC globally. Thus, this region was the focus of the majority of the studies.^3^ Various imaging modalities using AI have been used for OC screening and detection. For example, clinical photographs were used in various studies to demonstrate that suspected OSCC lesions could be differentiated automatically and easily by the application of algorithms.^10^^,^^26^^,^^27^ Al-Rawi et al. analyzed AI usage in the diagnosis of OC in 17 studies. They reported that ML was used in six studies and deep learning in the remainder. They concluded that deep learning was more precise than supervised ML for OC early diagnosis.^6^

A scoping review highlighted the effect that the variabilities of photographic images could have on the identification process of OC or OPMDs.^3^ Warin et al. conducted a study to develop an automated classification and detection system for OC screening. This study included 700 clinical oral photographs, consisting of 350 images of OSCCs and 350 images of normal oral mucosa. DenseNet121 was used for the classification model and faster R-CNN for the detection model. The study concluded that the DenseNet121 and faster R-CNN algorithm had the potential for the detection and classification of cancerous lesions.^20^

A multicenter study used simple smartphone probes with deep learning algorithms for OC screening. The screening was done in high-risk populations in inaccessible regions with limited infrastructure facilities, with a probe designed to access all parts of the oral cavity. The autofluorescence and polarization images from the probe were combined with a tabulation of risk factors, such as habits. The information was analyzed by deep learning-based algorithms, which then generated outputs for screening guidance.^28^, ^29^, ^30^

Optical coherence tomography (OCT) has been used in a few studies for diagnosis with AI. Studies indicated that the addition of a diagnostic algorithm to an OCT system would overcome the training requirements of the users concerning the reading of the OCT images. A low-cost OCT prototype was used to develop and evaluate an automated diagnostic algorithm linked to an image-processing application and user interface. Ilhan et al. reported that the automated cancer screening platform could differentiate between healthy and dysplastic/malignant tissues with 87% sensitivity and 83% specificity.^4^ Similarly, Ramezani et al. reported that AI algorithms rendered positive outcomes in the interpretation of OCT images of normal oral mucosa and precancerous and cancerous lesions. Automated OC screening by OCT requires the progression of AI algorithms for their interpretation; hence, a continuous data feed is needed to function as ground information.^31^

Tissue sections of head and neck cancers from different sites, such as the tongue, floor of the mouth, gingivae, alveolar ridge, mandible, soft palate, supraglottis, nose, maxillary sinus, parotid gland, and thyroid were evaluated in various studies.^32^, ^33^, ^34^ The role of ML techniques as a diagnostic tool for histology images in recognizing OSCC and a few OPMDs was highlighted in recent systematic reviews.^35^ García-Pola et al. reported that a few studies used exfoliative, liquid, scraped, and brush biopsies for cytological diagnosis.^3^ Mahmood et al. evaluated studies that used various ML approaches to differentiate specific histological features and compare alterations in the spatial architectural patterns to statistically distinguish benign and malignant lesions. They stated that unicentric small datasets could lead to a high risk of bias due to limited evidence. Therefore, they recommended that prospective studies with large samples and in multiple centers would best support the medical practice.^36^

According to Mahmood et al., algorithm training mainly included histology whole-slide and radiological imaging. The increasing use of digital slide scanners in pathology laboratories and the advent of radiomics have widened the scope. Sultan et al. reported that AI had achieved admirable results compared to pathologists. Studies on OSCC digital histopathologic images demonstrated potential when the predictive models included both clinical and genomic data. When the skills of expert pathologists were combined with AI systems, superior results could be delivered with fewer diagnostic errors.^37^ A Cochrane review stated that none of the early diagnostic tests available at that time could replace a biopsy for an OC diagnosis.^38^ Chiesa-Estomba et al. analyzed eight studies and concluded that ML had the potential to considerably advance the field of OC research due to the ability of ML models to constantly learn with additional data.^39^

### AI in oral cancer prediction

In recent years, much research has been done in the field of OC and AI.^40^ Several studies reported that developing AI models could successfully predict the occurrence and recurrence of OC.^22^^,^^23^^,^^41^^,^^42^ AI has been used in cancer prognosis to predict lymph node metastasis and assess the risk of cancer. As mentioned previously, the prediction of the malignant transformation of OPMDs is crucial in the prevention/early diagnosis of OSCC.

Adeoye et al. successfully used time-to-event learning models to assess the risks of malignancy in oral leukoplakia and oral lichenoid lesions in 1098 subjects.^24^ Cancer biomarkers are directly produced by tumor cells or by non-tumor cells influenced by tumor tissue. Biomarker detection helps with the understanding of pathogenesis and cancer prognosis. As the use of cancer biomarkers in clinics is limited, AI's ability to analyze large quantities of data would help detect biomarkers accurately.^43^

The most common metastasis pathway for cancer is lymph node metastasis, which leads to a poor prognosis. A study by Bur et al. predicted the pathological lymph node metastasis accurately in patients with OSCC.^44^ Sun et al. reported that the most widely used imaging method for evaluating cervical lymph nodes was contrast-enhanced CT (CECT). They further noted that the prediction result was highly accurate in extranodal extension.^43^ Several studies were conducted to identify the proteins and peptides as predictors of lymph node metastasis.^43^ Sun et al. suggested that the proliferation status of tongue squamous cell carcinoma could be non-invasively predicted using AI in CECT prior to operation.^17^ Ilhan et al. reported that the introduction of numerous new omics technologies, such as genomics and proteomics, made it possible to collect vast quantities of cancer data. In OC and the omics research field, AI applications were used to develop prognostic prediction models, determine node involvement and human papillomavirus (HPV)-associated biomarkers, and detect transcriptomic and metabolite signatures. The clinical practice could thus be improved with advanced and combined clinicopathologic imaging features and omics data through an AI approach.^45^

AI allows automated learning without human adjudication. These models predict future events based on a current set of observations.^46^ AI is able to automate processes and combine variables at different levels and provide outcomes to guide clinical decision-making. Micromorphological features can be combined with geographical data, risk factors, varied signal intensity, and patterns to generate risk assessments.^4^ Kim et al. used a deep learning program to predict OC patients’ survival and found that the program gave a superior diagnostic performance. They concluded that AI-based anticipation prediction could provide satisfactory results.^41^ This concurs with the review by Khanagar et al., which reported that previous studies that used AI to predict OC yielded excellent results.^12^

### AI in oral cancer management

AI has been used to solve problems in planning head and neck cancer treatment, such as automated treatment planning, supportclinical decision support, intensity-modulated radiotherapy (IMRT) dose calculation, treatment outcome, and auto-segmentation for patients with cancer.^47^ Alabi et al. conducted a systematic review and stated that the deep learning techniques assisted the clinicians with informed decision-making, selection of treatment options, and improved management of OSCC.^48^ Treatment choice and prognosis of OC depend on early detection and cervical lymph node metastasis, and as such, AI systems may assist clinicians in the management of OC.^49^

### Limitations

The research information used in this review was limited to data retrieved from articles published in PubMed and Scopus databases only. As a result, the limitations inherent in those articles affected the detail of this review. These limitations included a limited amount of data available, the retrospective collection of data, image quality, the comparison of images taking into consideration that healthy tissue could be present with mucosal alterations, imbalances in the data used to train, the risk of missing data, the lack of studies collaborating with clinical diagnosis and biopsy findings, the task of automation, infrastructure requirements for data storage, building and training AI models, and patient privacy and ethical issues.^3^^,^^39^

### Implications for future research

Future studies in the field of AI and OC may be helpful in overcoming the present limitations. Radiomics is an emerging field of research using radiographic images and data characterization algorithms. Radiomics has helped extract features, such as intensity, shape, and surface texture from CT and MRI images of malignancy, which might go unnoticed by the human eye. Radiomics could become a valuable tool for oncologists to evaluate tumor metastatic potential, oncogene expression, and treatment response.^2^

## Conclusions

In recent years, the use of AI for the diagnosis and prognosis of diseases has evolved. Previous studies have proved that ML produces accurate results for OC detection. It assists clinicians in diagnostic processes and minimizes inadvertent errors. However, previous studies based on deep learning (neural network) provided more accuracy with the early detection of OC as compared to ML. AI presents the opportunity to develop new techniques combined with traditional approaches to improve the accuracy of detection of OC and OPMDs, as well as to predict the course of the precancerous/cancerous lesions from retrospective data. Future research could consider the innovation of data fusion algorithms combining various modalities, such as clinical, radiological, histological, and molecular assessments, to support the early diagnosis and outcome estimation of the disease.

## Author contributions

HS, AV: conceptualization; data curation; formal analysis; investigation; methodology; resources; writing - original draft; WZ, CZ: supervision methodology, review, and editing.

## Declaration of competing interest

None declared.

## Funding

Nil.
